# Changes in treatment and mortality of acute myocardial infarction in Estonian tertiary and secondary care hospitals in 2001 and 2007

**DOI:** 10.1186/1756-0500-5-71

**Published:** 2012-01-26

**Authors:** Mai Blöndal, Tiia Ainla, Toomas Marandi, Aleksei Baburin, Jaan Eha

**Affiliations:** 1 Department of Cardiology, University of Tartu, L. Puusepa 8, 51014 Tartu, Estonia; 2 Heart Clinic, Tartu University Hospital, L. Puusepa 8, 51014 Tartu, Estonia; 3 Centre of Cardiology, North Estonia Medical Centre Foundation, J. Sütiste tee 19, 13419 Tallinn, Estonia; 4 Quality Department, North Estonia Medical Centre Foundation, J. Sütiste tee 19, 13419 Tallinn, Estonia; 5 Department of Epidemiology and Biostatistics, National Institute for Health Development, Hiiu 42, 11619 Tallinn, Estonia

**Keywords:** Acute myocardial infarction, Treatment, Revascularisation, Mortality

## Abstract

**Background:**

High quality care for acute myocardial infarction (AMI) improves patient outcomes. Still, AMI patients are treated in hospitals with unequal access to percutaneous coronary intervention. The study compares changes in treatment and 30-day and 3-year mortality of AMI patients hospitalized into tertiary and secondary care hospitals in Estonia in 2001 and 2007.

**Results:**

Final analysis included 423 cases in 2001 (210 from tertiary and 213 from secondary care hospitals) and 687 cases in 2007 (327 from tertiary and 360 from secondary care hospitals). The study sample in 2007 was older and had twice more often diabetes mellitus. The patients in the tertiary care hospitals underwent reperfusion for ST-elevation myocardial infarction, cardiac catheterization and revascularisation up to twice as often in 2007 as in 2001. In the secondary care, patient transfer for further invasive treatment into tertiary care hospitals increased (*P *< 0.001). Prescription rates of evidence-based medications for in-hospital and for outpatient use were higher in 2007 in both types of hospitals. However, better treatment did not improve significantly the short- and long-term mortality within a hospital type in crude and baseline-adjusted analysis. Still, in 2007 a mortality gap between the two hospital types was observed (*P *< 0.010).

**Conclusions:**

AMI treatment improved in both types of hospitals, while the improvement was more pronounced in tertiary care. Still, better treatment did not result in a significantly lower mortality. Higher age and cardiovascular risk are posing a challenge for AMI treatment.

## Background

In the last decade, Estonia has reported one of the highest rates of mortality due to ischemic heart diseases in Europe [1]. At the same time, as in other East European countries, the health care system in Estonia has undergone considerable changes. Lead by the Estonian Society of Cardiology, much effort has been made to improve the quality of care for acute myocardial infarction (AMI) patients through better application of the diagnosis and treatment guidelines [2-7].

One of the main priorities has been to increase access to percutaneous coronary interventions (PCI) and to enable more ST-segment elevation AMI (STEMI) patients receive reperfusion, including primary PCI. According to a recent study, the rates of primary PCI in Estonia are now comparable to those in such Nordic countries as Norway and Denmark [8].

Previous studies have mainly focused on the overall changes in the treatment and mortality of AMI patients [9-14]. Changes in different types of hospitals with unequal availability of coronary intervention facilities have received little attention. Still, such information is crucial in a country with limited health care resources aiming to provide equal care for all AMI patients.

This study aimed to determine the changes in in-hospital treatment and 30-day mortality and 3-year mortality of AMI patients hospitalized into tertiary and secondary care hospitals in Estonia in 2001 and 2007.

## Methods

**Figure 1:**
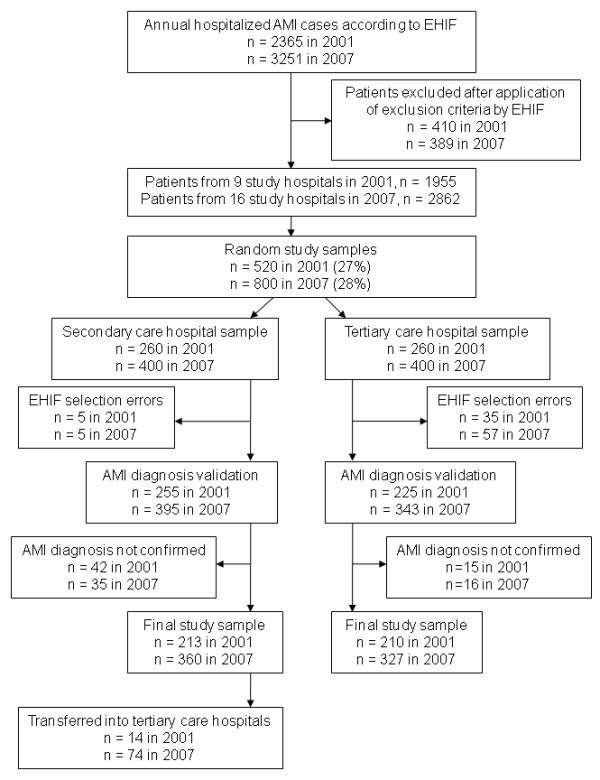
**Formation of the study samples in 2001 and 2007**. AMI, acute myocardial infarction; EHIF, Estonian Health Insurance Fund.

We conducted a retrospective cross-sectional study based on patient records. The formation of the study samples is presented in Figure 1. The list of AMI cases (main diagnosis code I21-I22 according to the International Statistical Classification of Diseases and Related Health Problems 10^th ^revision [15]) hospitalized from January 1 to December 31, 2001 and 2007, was obtained from the database of the Estonian Health Insurance Fund (EHIF). The Estonian health insurance system is a social insurance relying on the principle of solidarity and of the 1.3 million inhabitants about 95% are insured. Consistency in reporting to the EHIF database and the validity of the data has been established [16].

The EHIF applied the following exclusion criteria for case selection: (1) patients who were not first hospitalized into one of the study hospitals; (2) patients who were re-admitted with AMI within 28 days of the first admission; (3) patients whose length of hospital stay was less than 3 days if they were discharged alive and were not transferred to another hospital, which made the diagnosis of AMI unlikely.

In 2001, according to the EHIF database, 2365 AMI cases were hospitalized during the study period in Estonia. Management of AMI patients was shared among 27 Estonian hospitals with a different number of beds. As we aimed to evaluate the management of AMI patients in hospitals that treat the major proportion of annual AMI cases, the study included 9 hospitals: 2 tertiary PCI-capable (only during working hours) and 7 secondary care hospitals. In the secondary care hospitals the number of annual AMI cases ranged from 7 to 165 cases and the tending physicians were mainly anesthesiologists or internists and in some hospitals also cardiologists. After the application of the exclusion criteria by the EHIF, 1955 cases remained, out of which a random sample of 520 cases (27%) was formed. The sampling was performed in clusters according to the hospitals involved.

In 2007, 3251 AMI cases were hospitalized in Estonia. Due to the changes in the hospital network only 20 hospitals admitted AMI patients in 2007. The study included 16 hospitals that treated the major proportion of annual AMI cases: 2 tertiary 24/7 PCI-capable and 14 secondary care hospitals (one hospital had PCI availability during working hours). The tertiary care hospitals were the same in both time periods. In the secondary care hospitals the number of annual AMI cases ranged from 16 to 267 cases and the tending physicians were mainly anesthesiologists or internists and in some hospitals also cardiologists. After the application of the exclusion criteria by the EHIF, 2862 cases remained, out of which a random sample of 800 cases (28%) was formed. The sampling was performed in clusters according to the hospitals involved.

### Data collection

Data from medical records were retrospectively abstracted by study experts according to a standardized data collection form. Most of the experts were certified cardiologists and all had received additional training on data collection for this study. Every case was reviewed by one expert. Data quality was monitored by random re-abstractions for determining the causes of discrepancies and followed by retraining of experts. The abstracted data included: (1) patient baseline characteristics; (2) time of symptom onset; (3) cardiac catheterization, revascularization, and non-invasive cardiac testing during index hospitalization; (4) in-hospital and discharge medications; (5) in-hospital mortality. The date of death during 3-year follow-up was obtained from the Estonian Population Register by the EHIF. The study was approved by the Research Ethics Committee of the University of Tartu (reference number 196/T-1).

As we aimed to assess the quality of care of the first hospital where the patient was hospitalized, the data collection stopped when the patients were transferred from a secondary care to a tertiary care hospital.

### Statistical analysis

Categorical variables were expressed as frequencies and percentages, and continuous variables as means and standard deviations (SD), or as medians and interquartile ranges (IQR). To compare the patients admitted into the tertiary and secondary care hospitals in 2001 and 2007 in respect to baseline characteristics, in-hospital management and discharge prescription rates of medications, the Chi-Square or the Fisher's exact test for categorical variables and the *t*-test for two independent samples or the Wilcoxon-Mann-Whitney test for continuous variables were used.

The outcome was expressed as 30-day and 3-year mortality. The crude and baseline-adjusted [age, sex, AMI subtype (STEMI or non-STEMI), diabetes mellitus, arterial hypertension, previous AMI, previous heart failure] hazard ratios (HR) of mortality for patients admitted into the tertiary and secondary care hospitals in 2001 vs 2007 were estimated with Cox's proportional hazards regression. The ratio with the 95% confidence interval (CI) was presented as the ratio of the rate in 2007 to the rate in 2001 in the tertiary or secondary care hospitals.

The experts found that the documentation of smoking status and lipid profiles in medical records was incomplete in 2001 and 2007 (percentage of missing values ranged from 14.5-48.8) and those variables were not further analyzed in the patient characteristics of the results section.

Two-sided *P *values ≤ 0.05 were considered statistically significant. All analyses were performed using the Stata statistical software version 11.

## Results

Out of the random study samples (n = 520 in 2001, n = 800 in 2007), 40 (7.8%) cases in 2001 and 62 (7.8%) cases in 2007 were excluded from the analysis because of selection errors that arose in the forming of the study population by the EHIF (the study sample included cases that were not first hospitalized into one of the study hospitals).

Among the remaining cases, the diagnosis of AMI was confirmed for the following patients: 93.3% in 2001 and 95.3% in 2007 (*P *= 0.304) in the tertiary care hospitals; 83.5% in 2001 and 91.1% in 2007 (*P *= 0.003) in the secondary care hospitals. The diagnosis of AMI in both study periods was based on the redefinition document of myocardial infarction published in 2000 [2].

The final study samples included 423 cases in 2001 (210 from the tertiary and 213 from the secondary care hospitals) and 687 cases in 2007 (327 from the tertiary and 360 from the secondary care hospitals).

### Patient characteristics

In the study sample of the tertiary care hospitals, the proportion of men, patients with STEMI and diabetes mellitus was higher in 2007 compared to 2001 (Table 1). The patients of the study sample in the secondary care hospitals were considerably older, had more often diabetes mellitus, and hypertension in 2007 compared to 2001.

**Table 1:** Baseline characteristics of patients hospitalized into tertiary and secondary care hospitals in Estonia in 2001 and 2007

	Tertiary care hospitals			Secondary care hospitals		
	**Year 2001**	**Year 2007**	***P***	**Year 2001**	**Year 2007**	***P***

	**n = 210**	**n = 327**		**n = 213**	**n = 360**	

Mean age (SD), yrs	68.3 (12.7)	69.7 (12.0)	0.189	68.3 (12.4)	71.8 (11.4)	0.001
≥ 75 yrs, %	30.9	37.0	0.150	34.3	45.3	0.010
Men, %	66.7	58.1	0.047	52.1	51.9	0.969
STEMI, %	61.9	49.5	0.005	59.6	51.4	0.056
Diabetes mellitus, %	19.1	27.2	0.028	16.4	31.1	< 0.001
Arterial hypertension, %	70.0	70.0	0.914	57.3	75.8	< 0.001
Previous AMI, %	29.5	29.4	0.967	23.9	27.2	0.384
Previous heart failure, %	27.1	28.1	0.802	26.8	31.7	0.215
Time to presentation (hrs), %						
≤ 3	50.0	42.5	0.215	33.3	30.8	0.878
3-12	24.3	24.8		25.8	25.3	
> 12-24	9.5	9.8		12.2	12.2	
> 24	16.2	22.9		28.6	31.7	

AMI, acute myocardial infarction; SD, standard deviation; STEMI, ST-segment elevation myocardial infarction

### Management during hospitalization

Prescription rates of evidence-based medications were higher in 2007 in both types of hospitals (Table 2).

**Table 2:** Management during hospitalization in tertiary and secondary care hospitals in Estonia in 2001 and 2007

	Tertiary care hospitals			Secondary care hospitals		
	**Year 2001**	**Year 2007**	***P***	**Year 2001**	**Year 2007**	***P***
	**%**	**%**		**%**	**%**	

	**n = 210**	**n = 327**		**n = 213**	**n = 360**	

Medications						
Aspirin	87.1	94.2	0.004	88.3	86.4	0.428
Clopidogrel/ticlopidine	17.1	61.5	< 0.001	0.0	10.6	NA
Anticoagulants	89.0	93.0	0.092	85.4	92.8	0.007
Glycoprotein IIb/IIIa inh	12.4	38.8	< 0.001	0.5	3.1	0.037
Beta-blockers	79.5	82.6	0.395	76.1	77.8	0.707
Nitrates	92.4	78.9	< 0.001	96.7	85.6	< 0.001
ACEI/ARB	70.5	74.9	0.267	37.1	62.2	< 0.001
Statins	26.7	67.9	< 0.001	5.6	30.8	< 0.001
Cardiac catheterization	35.7	78.3	< 0.001	0.0	6.7	NA
Revascularisation	27.6	64.2	< 0.001	0.0	4.2	NA
PCI	22.4	61.5	< 0.001	0.0	4.2	NA
CABG	5.2	3.7	0.381	0.0	0.0	NA
Reperfusion for STEMI	42.3	64.2	< 0.001	44.1	34.1	0.073
Thrombolysis	35.4	7.4	< 0.001	44.1	34.1	0.073
Primary PCI	6.9	56.8	< 0.001	0.0	0.0	NA
Echocardiography	81.9	85.3	0.292	52.1	51.9	0.969
Stress-testing	19.0	1.8	< 0.001	8.0	2.6	0.023

ACEI, angiotensin-converting enzyme inhibitors; ARB, angiotensin II receptor blockers; CABG, coronary artery bypass grafting; NA, not applicable; PCI, percutaneous coronary intervention; STEMI, ST-segment elevation myocardial infarction

The patients of the tertiary care hospitals underwent reperfusion for STEMI, cardiac catheterization and revascularisation up to twice as often in 2007 as in 2001. The reperfusion rates for STEMI did not change significantly in the secondary care hospitals.

Fourteen (6.6%) cases in 2001 and 74 (20.6%) cases in 2007 were transferred from a secondary to a tertiary care hospital for further management (*P *< 0.001).

### Medications for outpatient use

In order to analyze the prescription rates of medications for outpatient use in the tertiary care hospitals, we excluded the patients who died during hospital stay [30 (14.3%) in 2001 and 37 (11.3%) in 2007]. In the secondary care hospitals, we excluded those who died during hospital stay [36 (16.9%) in 2001 and 62 (17.2%) in 2007], or were transferred into tertiary care hospitals [14 (6.6%) in 2001 and 74 (20.6%) in 2007].

The prescription rates of evidence-based medications for outpatient use increased in all studied drug groups in both hospital types in 2007 compared to 2001 (except for aspirin in the secondary care hospitals) (Table 3). At the same time, there was a decrease in the use of nitrates in both types of hospitals.

**Table 3:** Medications prescribed for outpatient use in tertiary and secondary care hospitals in Estonia in 2001 and 2007

	Tertiary care hospitals			Secondary care hospitals		
**Medications**	**Year 2001** **%**	**Year 2007** **%**	***P***	**Year 2001** **%**	**Year 2007** **%**	***P***

	**n = 180**	**n = 290**		**n = 163**	**n = 224**	

Aspirin	85.1	93.1	< 0.001	79.8	82.6	0.776
Clopidogrel/ticlopidine	18.3	65.2	< 0.001	0.6	10.3	< 0.001
Beta-blockers	71.3	80.0	0.035	68.7	80.8	0.032
ACEI/ARB	66.7	77.6	0.007	37.4	69.2	< 0.001
Statins	31.5	73.4	< 0.001	14.5	37.1	< 0.001
Nitrates	61.9	22.1	< 0.001	85.3	58.0	< 0.001

ACEI, angiotensin-converting enzyme inhibitors; ARB, angiotensin II receptor blockers

### Mortality

**Figure 2:**
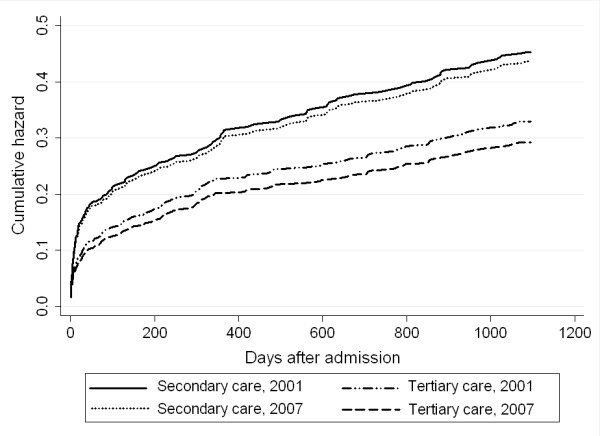
**Baseline-adjusted 3-year cumulative mortality hazards of patients hospitalized into tertiary and secondary care hospitals in Estonia in 2001 and 2007**.

In crude and baseline-adjusted analysis there were no significant differences in the 30-day mortality and 3-year mortality within the two hospital types in 2007 compared to 2001 (Table 4 and Figure 2).

**Table 4:** Mortality of patients hospitalized into tertiary and secondary hospitals in Estonia in 2001 and 2007

	Year 2001	Year 2007			
**Mortality**	**%**	**%**	***P***	**HR (95% CI)**	**Adjusted HR (95% CI)^a^**

30-day					
Tertiary care hospitals	17.6	13.2	0.156	0.71 (0.44-1.15)	0.69 (0.42-1.13)
Secondary care hospitals	20.2	22.5	0.516	1.17 (0.78-1.77)	1.01 (0.66-1.55)
3-year					
Tertiary care	35.7	32.7	0.475	0.90 (0.66-1.22)	0.90 (0.65-1.23)
Secondary care hospitals	38.5	45.3	0.113	1.24 (0.94-1.64)	1.03 (0.77-1.37)

CI, confidence interval; HR, hazard ratio

^a ^Adjusted for age, sex, AMI subtype (STEMI or non-STEMI), diabetes mellitus, arterial hypertension, previous acute myocardial infarction and previous chronic heart failure.

Comparison of mortality between the two hospital types in 2001 showed no significant differences (results not presented). However, in 2007, the 30-day mortality was significantly lower in the tertiary care hospitals than in the secondary care hospitals in crude (HR 0.54; 95% CI 0.36-0.81) and baseline-adjusted analysis (HR 0.61; 95% CI 0.41-0.93). Also after 3 years mortality in the tertiary care hospitals remained lower in crude (HR 0.66; 95% CI 0.51-0.85) and baseline-adjusted analysis (HR 0.73; 95% CI 0.57-0.95).

## Discussion

This country-wide study shows that between 2001 and 2007, the treatment of AMI patients improved in the tertiary and secondary care hospitals in Estonia, although the rates of reperfusion and revascularization increased mainly in the tertiary care setting. At the same time, improvements in the treatment did not translate into significantly lower mortality within the hospital types even after accounting of the differences in the baseline characteristics. Instead, in 2007, a marked mortality gap can be observed between the tertiary and the secondary care hospitals.

The positive trend in AMI treatment during hospitalization has also been reported by previous studies [9-14,17-19]. The observed improvements in Estonia are probably connected to better access to coronary intervention facilities, the release of new European and Estonian guidelines during the study period, and the launching of several training programs by the Estonian Society of Cardiology [3-7].

Still, in 2007, the mean use of beta-blockers and ACEI/ARB during hospitalization and for outpatient use remained below 83%, while the rates in the secondary care hospitals were even lower. The use of statins in the secondary care hospitals causes even more concern as although the rates increased markedly during the study period, their use is still below 38%. The fact that the quality of care of AMI patients in the secondary care hospitals lags behind that in their tertiary care counterparts may be due to the known slower implementation of guideline-recommended medications in secondary care hospitals [20]. As the study demonstrated, patients in the secondary care hospitals tend to be older and have more co-morbidities, which may influence management decisions.

Better access to invasive coronary care facilities and recent research findings contributed to the wider use of PCI in the tertiary care hospitals. At the same time, in the secondary care hospitals transfer for further cardiac testing and revascularisation into tertiary care hospitals increased. Still, the transfer rates were low compared to previous studies [21,22]. Although the mean age of transferred patients increased, transferred patients still tended to be younger than the non-transferred patients (data not presented).

While in the tertiary care hospitals reperfusion rates increased and primary PCI became the preferred method of reperfusion for STEMI patients, then in the secondary care hospitals reperfusion rates showed little change. It is possible that STEMI patients are usually transferred before receiving medical reperfusion. Still, a previous study revealed that the reasons for not receiving reperfusion may be unknown in up to 45% of cases [23].

Several studies have demonstrated that closer adherence to published guidelines for AMI management results in improved short-and long-term outcomes and this even despite the growing prevalence of risk factors (older age, history of hyperlipidaemia and diabetes mellitus) at presentation [10-14,16,24-26]. Although our study demonstrates a marked improvement in the treatment quality for AMI patients in 2007 compared to 2001, especially in tertiary care hospitals, it fails to show a significant decrease in 30-day and 3-year mortality. As the study sample of the secondary care hospitals demonstrated, this may largely be due to the higher age and cardiovascular risk among the study samples in 2007. For instance, the rates of diabetes had almost doubled in both types of hospitals. In order to further clarify this issue, we performed a sub-analysis to compare the baseline characteristics, quality of care provided, and mortality separately among patients < 75 and ≥ 75 years in 2001 and 2007 in tertiary care hospitals (data not presented). On the basis of this analysis we may hypothesize that in tertiary care hospitals the reason why short-and long-term mortality has not improved despite better overall quality of care is that firstly the improvement is more targeting those younger and healthier and secondly the rate of patients over 75 years with more co-morbidities has increased.

Interesting findings of the study were the differences in short and long-term mortality between the two hospital types in 2007. This can probably be explained by a combined effect of improved management possibilities in the tertiary care hospitals and different patient baseline characteristics in the two hospital types. The patients admitted to the secondary care hospitals are more likely to be older and have a higher cardiovascular risk. Moreover, as the elderly often present with atypical symptoms and have a greater burden of cardiac and non-cardiac co-morbidities, physicians are more reluctant to treat them aggressively, the more so when the outcomes of interventions and surgery may be poorer [27,28].

## Limitations

Firstly, this study includes only random study samples which may not represent the true patient populations. As a consequence, the practice patterns observed in the present study may not be representative of the general AMI patient population. Secondly, the sample of 2007 included patients from seven secondary care hospitals that were not included in 2001 (accounting for 25% of the cases from the secondary care hospitals). This corresponds to the study principle of including hospitals that treat the major proportion of annual AMI cases. Limiting the analysis to only those secondary care hospitals that participated both in 2001 and 2007 produced similar treatment and mortality results (data not presented). Thirdly, the study did not capture information on co-morbid conditions such as chronic kidney disease and cancer, which may have precluded the use of certain pharmacologic and interventional treatments. Fourthly, owing to the study design, we were not fully able to collect information on smoking and lipid profiles and hence we could not account for these variables in mortality analysis.

## Conclusions

In Estonia, both types of hospitals underwent considerable improvement in AMI treatment between 2001 and 2007. Still, changes were more pronounced in the tertiary care setting, especially with respect to the use of reperfusion and revascularisation. Low rates of reperfusion and transfer for PCI clearly need to be addressed in the secondary care hospitals. The improvement in treatment did not result in a significant change in short-and long-term mortality in either hospital type. Higher age and cardiovascular risk are posing a challenge for AMI management.

In addition to addressing the gaps in the quality of AMI treatment across Estonian hospitals, there is a need for high-quality data from a prospective nationwide acute myocardial infarction register for continuous follow-up of AMI treatment and outcomes.

## Competing interests

The authors declare that they have no competing interests.

## Authors' contributions

MB, TA, TM, AB, and JE participated in the design of the study and in writing the manuscript. MB and AB performed the statistical analysis. All authors read and approved the final manuscript.

## Authors' information

MB, TA, TM, and JE are the members of the working group of Acute Coronary Syndromes of the Estonian Society of Cardiology as well as the members of the Scientific Board of the Estonian Myocardial Infarction Registry.
